# Binding of a [2Fe–2S] cluster drives dimerization of ferric uptake regulator (Fur) in *Escherichia coli*

**DOI:** 10.1016/j.jbc.2025.110702

**Published:** 2025-09-11

**Authors:** Fatemeh Najafi, Aidan G. Purcell, Finbar H. Homes, Huangen Ding

**Affiliations:** Department of Biological Sciences, Louisiana State University, Baton Rouge, Louisiana, USA

**Keywords:** iron homeostasis, ferric uptake regulator, Fur homodimer and monomer, iron–sulfur cluster, [2Fe-2S] cluster-bound Fur, Zn(II)-bound Fur, IscU.

## Abstract

The ferric uptake regulator (Fur) is a global transcription factor that reversibly binds an iron–sulfur [2Fe–2S] cluster *via* the cysteine residues (site 3) in response to elevation of intracellular free Fe content in *Escherichia coli*. Here, we report that when *E. coli* Fur is expressed in *E. coli* cells grown in M9 medium supplemented with Fe or zinc (Zn), purified Fur binds a [2Fe–2S] cluster or Zn(II), respectively. While apo-form Fur is a monomer and has no DNA-binding activity, both the [2Fe–2S] cluster–bound Fur and the Zn(II)-bound Fur are homodimers and have a similar binding activity for the DNA sequence known as the Fur-box. The inductively coupled mass spectrometry analyses show that the purified [2Fe–2S] cluster–bound Fur homodimer binds only one [2Fe–2S] cluster per monomer and no other transition cations, and that the Zn(II)-bound Fur homodimer binds only one Zn(II) per monomer. The site-directed mutagenesis studies reveal that Fur binds the [2Fe–2S] cluster or Zn(II) at the same binding site (site 3) *via* the cysteine residues. While deletion of the Fe–S cluster assembly scaffold protein IscU prevents the [2Fe–2S] cluster assembly in Fur, deletion of IscU has no effect on the Zn(II) binding in Fur. Furthermore, the addition of Zn(II) effectively inhibits the [2Fe–2S] cluster binding in Fur in *E. coli* cells grown in M9 medium. The results suggest that *E. coli* Fur dimerizes upon the binding of a [2Fe–2S] cluster at site 3 and that Zn(II) competes with the [2Fe–2S] cluster binding in Fur and disrupts the regulation of intracellular Fe homeostasis.

In *Escherichia coli* and many other bacteria, intracellular iron (Fe) homeostasis is primarily regulated by the ferric uptake regulator (Fur), a global transcription factor that controls expression of over 158 genes responsible for intracellular Fe transportation and storage, energy metabolism, oxidative stress, and bacteria virulence (1, 2, 3). Since the discovery of Fur in 1980s (4), it had been assumed that Fur binds a corepressor Fe(II) to regulate intracellular Fe homeostasis (5, 6, 7), because purified Fur can bind various divalent cations, including Fe(II), zinc (Zn(II)), cobalt (Co(II)), and manganese (Mn(II)) with dissociation constants ranging from 1.2 μM to 55 μM *in vitro* and become an active repressor to bind the DNA sequence known as Fur-box (4, 8, 9). Crystallographic studies of *E. coli* Fur (10) and its homologs from other bacteria (11, 12, 13, 14, 15) revealed that Fur proteins may exist as a homodimer or a tetramer, with each monomer containing three metal-binding sites: site 1 (coordinated by His-87, Asp-89, Glu-108, and His-125) is located within the dimerization domain; site 2 (coordinated by His-33, Glu-81, His-88, and His-90) connects the DNA-binding domain and the dimerization domain; and site 3 (coordinated by Cys-93 and Cys-96) is at the C-terminal end of the dimerization domain (10, 12). In the crystal structures, Fur often binds Zn(II) after reconstitution *in vitro* (11, 12, 13, 14). When *E. coli* Fur is expressed in *E. coli* cells grown in LB medium, purified Fur binds a tightly bound Zn(II) and a loosely bound Zn(II) (10, 16, 17). Chemical modification and mass spectrometry (MS) analysis demonstrated that *E. coli* Fur binds the tight Zn(II) at site 3 *via* the cysteine residues and forms a homodimer (18, 19). It is worth noting that the *Francisella tularensis* Fur is able to bind one Fe(II) or Mn(II) at site 2 and one Zn(II) at site 3 after reconstitution with Fe(II) or Mn(II) *in vitro*, respectively (15). Similarly, the *E. coli* Fur can also bind Fe(II) at site 2 and Zn(II) at site 3 after *in vitro* reconstitution (20). To the best of our knowledge, the Fe(II)-bound Fur had never been identified in *Escherichia coli* or any other bacteria. It was also reported that the exposure of *E. coli* Fur to nitric oxide (NO) produces the Fur-bound dinitrosyl Fe complex (21, 22), arguing that *E. coli* Fur binds a mononuclear Fe. However, the formation of dinitrosyl Fe complex in proteins by NO does not necessarily require a mononuclear Fe center. In fact, modification of iron–sulfur (Fe–S) clusters in proteins by NO will also readily produce the protein-bound dinitrosyl Fe complex (23, 24).

In searching for the proposed “Fe(II)-bound” Fur, we expressed *E. coli* Fur in the *E. coli* mutant cells with an elevated intracellular free Fe content in LB medium (25) or in wildtype *E. coli* cells grown in M9 medium supplemented with Fe (26) and found that purified Fur has a bright red color. By combining the UV–visible absorption, electron paramagnetic resonance, and Mössbauer spectroscopy approaches, we demonstrated that *E. coli* Fur binds a [2Fe–2S] cluster (not a mononuclear Fe) in response to elevation of intracellular free Fe content in *E. coli* cells (25, 26, 27). The whole cell Mössbauer spectroscopy measurements further showed that Fur binds an oxidized [2Fe–2S] cluster in *E. coli* cells (28). Additional studies indicated that *E. coli* Fur binds the [2Fe–2S] cluster *via* the cysteine residues (at site 3) (25), and that the [2Fe–2S] cluster assembly in Fur is enzymatically catalyzed by the Fe–S cluster assembly machinery in *E. coli* cells (29). Furthermore, the binding of the [2Fe–2S] cluster in Fur appears to be highly conserved, as the Fur homologs from *Haemophilus influenzae*, *Vibrio cholerae*, and *Helicobacter pylori* also bind a [2Fe–2S] cluster in the *E. coli* cells with an elevated intracellular free Fe content (30). More recently, an Fe–S cluster–bound *Acidithiobacillus ferrooxidans* Fur has been reported (31).

Here, we determine that when *E. coli* Fur is expressed in *E. coli* cells grown in M9 medium supplemented with Fe(II) or Zn(II), Fur binds a [2Fe–2S] cluster or Zn(II), respectively. The binding of the [2Fe–2S] cluster, like the binding of the Zn(II) (10, 16, 17, 18, 19), drives dimerization of Fur and turns on the Fur-box binding activity. The inductively coupled mass spectrometry (ICP–MS) measurements show that the purified [2Fe–2S] cluster–bound Fur homodimer binds only one [2Fe–2S] cluster per monomer with no other transition cations and that the Zn(II)-bound Fur homodimer binds only one Zn(II) per monomer. Site-directed mutagenesis studies further reveal that Fur binds the [2Fe–2S] cluster or the Zn(II) at the same binding site (site 3) *via* the cysteine residues. While deletion of the Fe–S cluster assembly protein IscU (32) prevents the [2Fe–2S] cluster assembly in Fur (29), deletion of IscU does not affect the Zn(II) binding in Fur and dimerization of Fur in *E. coli* cells. Furthermore, the addition of Zn(II) effectively blocks the [2Fe–2S] cluster binding in Fur in *E. coli* cells, suggesting that Zn(II) can compete for the [2Fe–2S] cluster binding in Fur and disrupt the regulation of the Fur-mediated intracellular Fe homeostasis.

## Results

### The [2Fe–2S] cluster–bound *E. coli* Fur is a homodimer

**Figure 1:**
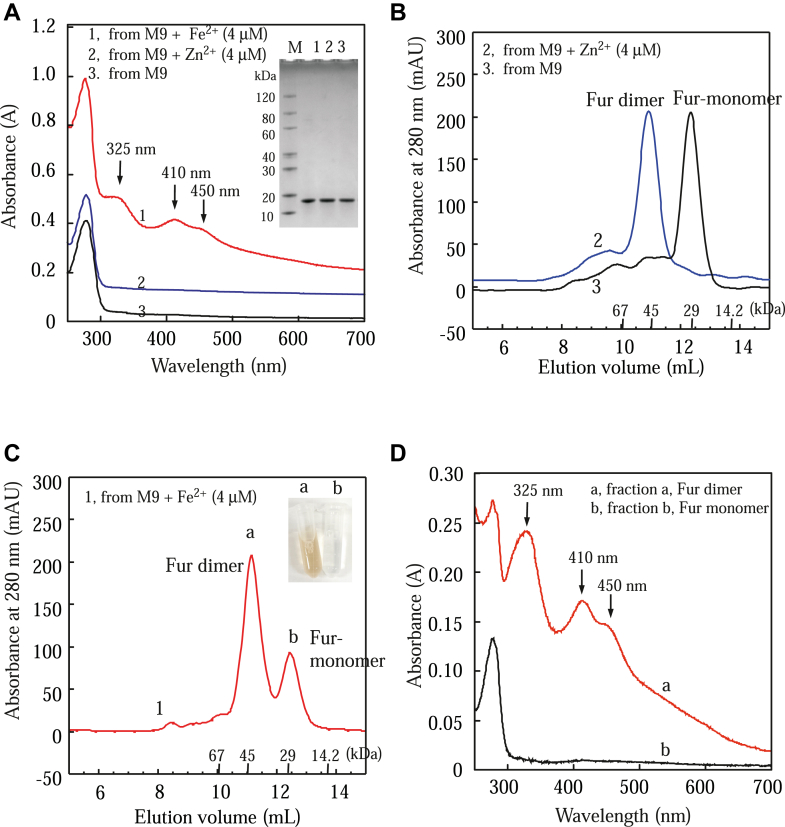
**The [2Fe-2S] cluster–bound *Escherichia coli* Fur is a homodimer.***A,* UV–visible absorption spectra of purified *E. coli* Fur proteins. *E. coli* Fur was expressed in wildtype *E. coli* cells (MC4100) grown in M9 medium supplemented with Fe(NH_4_)_2_(SO_4_)_2_ (4 μM) (spectrum 1), ZnSO_4_ (4 μM) (spectrum 2), or no addition (spectrum 3). Purified proteins (70 μM) were dissolved in buffer containing NaCl (500 mM) and Tris (20 mM, pH 8.0). Each spectrum was shifted by an absorbance of 0.1 for clarity. The inseted photograph is the SDS-PAGE gel of purified proteins. Lane M, the PAGE-MASTER protein markers (GenScript Co); lanes 1, 2, and 3, Fur proteins purified from *E. coli* cells grown in M9 medium supplements with Fe(NH_4_)_2_(SO_4_)_2_, ZnSO_4_, or no addition, respectively. *B,* gel filtration analysis of purified Zn-bound Fur and apo-form Fur. About 0.5 ml of purified Fur protein was loaded onto the gel filtration column (Superdex 75 10/300GL) (Cytiva Co) and eluted with the buffer containing NaCl (500 mM) and Tris (20 mM, pH 8.0) at a flow rate of 0.8 ml/min at room temperature. Trace 1, *E. coli* Fur (70 μM) purified from the *E. coli* cells grown in M9 medium supplemented with ZnSO_4_ (4 μM). Trace 2, *E. coli* Fur (70 μM) purified from the *E. coli* cells grown in M9 medium only. The elution volumes of the molecular weight markers of 67 kDa, 45 kDa, 29 kDa, and 14.2 kDa are shown at the *x*-axis. *C,* gel filtration analysis of purified [2Fe–2S] cluster–bound Fur. *E. coli* Fur (70 μM) purified from the *E. coli* cells grown in M9 medium supplemented with Fe(NH_4_)_2_(SO_4_)_2_ (4 μM). About 0.5 ml of purified Fur protein was loaded onto the gel filtration column as described. The inserted photograph is the image of the eluted fractions of peaks a and b. *D,* UV–Visible absorption spectra of eluted Fur fractions. Spectrum a (*red*), eluted fraction a (Fur dimer) containing a [2Fe–2S] cluster. Spectrum b (*black*), eluted fraction b (Fur monomer) containing no [2Fe–2S] clusters. Both Fur homodimer and monomer are shown at the concentration of 30 μM Fur monomer. The data are representatives of three independent experiments. Fe–S, iron–sulfur; Fur, ferric uptake regulator.

M9 medium is known to be Fe deficient (containing ∼0.05 μM total Fe) (33). Addition of exogenous Fe in growth medium is often used to elevate intracellular-free Fe content in *E. coli* cells (34). When M9 medium is supplemented with 2 μM Fe citrate, the intracellular Fe content per dry weight of *E. coli* cells at the exponential growth phase increases from 0.005% to 0.015% (35). Further increase of Fe citrate (up to 128 μM) in M9 medium does not increase the intracellular Fe content of the cells because of regulation of intracellular Fe homeostasis *via* Fur (35). Here, *E. coli* Fur was expressed in wildtype *E. coli* cells grown in M9 medium supplemented with FeNH_4_(SO_4_)_2_ (4 μM), ZnSO_4_ (4 μM), or with no addition of metals. Purified Fur proteins were subjected to UV–visible absorption measurements. Figure 1*A* shows that Fur purified from the *E. coli* cells grown in M9 medium supplemented with Fe(II) (4 μM) had a bright red color with absorption peaks at 325 nm, 410 nm, and 450 nm (spectrum 1), as reported previously (26). The electron paramagnetic resonance and Mössbauer spectroscopic measurements demonstrate that the purified red Fur binds a [2Fe–2S] cluster (25, 28). When *E. coli* Fur was expressed in *E. coli* cells grown in M9 medium supplemented with Zn(II) (4 μM) (spectrum 2) or with no addition of metals (spectrum 3), purified Fur proteins had no color and no absorption peaks in the visible region (Fig. 1*A*).

Purified Fur proteins were then subjected to gel filtration analyses. Figure 1*B* shows that the Fur purified from the *E. coli* cells grown in M9 medium with no addition of transition metals was a monomer with a molecular weight of about 23 kDa, consistent with the previous report showing that apo-form Fur is a monomer (10, 17, 19). On the other hand, the Fur purified from the *E. coli* cells grown in M9 medium supplemented with Zn(II) was a homodimer with a molecular weight of about 45 kDa (Fig. 1*B*), in agreement with the previous notion that the Zn(II)-bound Fur is a homodimer (10, 16, 17, 18, 19). When the Fur purified from the *E. coli* cells grown in M9 medium supplemented with Fe(II) was subjected to gel filtration analyses (Fig. 1*C*), two major peaks were eluted: peak a, at a molecular weight of about 45 kDa (Fur homodimer), had a bright red color; peak b, at a molecular weight of about 23 kDa (Fur monomer), had no color (Fig. 1*C*, *inset*). Thus, the Fur protein purified from the *E. coli* cells grown in M9 medium supplemented with Fe(II) had two populations: the [2Fe–2S]-bound Fur homodimer (peak a) and the apo-form Fur monomer (peak b), as reported previously (26). When the Fur homodimer or monomer was reinjected into the gel filtration column, only the Fur homodimer or monomer was eluted, respectively, suggesting that the Fur homodimer was stable in solution.

The purified Fur homodimer and apo-form monomers purified from Figure 1*C* were then subjected to the UV–visible absorption measurement. Figure 1*D* shows that the Fur homodimer (fraction 21) had an absorption spectrum of a typical [2Fe–2S] cluster with the absorption peaks at 325 nm, 410 nm, and 450 nm, whereas the apo-form Fur did not have any absorption peaks in the visible region. The CD measurements (Fig. S1) showed that both Fur homodimer and monomer had similar secondary structures, although the subtle differences of the overall CD spectrum between the Fur homodimer and monomer were evident, indicating that the Fur conformation is altered upon the binding of a [2Fe–2S] cluster.

The [2Fe–2S] cluster–bound Fur homodimer, the apo-form Fur monomer, and the Zn(II)-bound Fur homodimer were subjected to the Fe and acid-labile sulfide content analysis according to the Fischer’s method (36) and the Siegel’s method (37), respectively. The Zn(II) was tightly bound in Fur and could only be released by the urea (6 M) denaturation as described (16). The Fe and Zn contents in purified Fur samples were also determined using the ICP–MS (Georgia University, Center for Applied Isotope Studies). We found that the purified [2Fe–2S] cluster–bound Fur homodimer contained 1.86 ± 0.30 Fe atoms, 1.60 ± 0.40 sulfide atoms, and less than 0.1 Zn atoms per monomer (n = 3), suggesting that the [2Fe–2S] cluster–bound Fur homodimer binds only one [2Fe–2S] cluster and no other transition cations. The apo-form Fur monomer contained less than 0.1 Fe, sulfide, or Zn atoms per monomer, consistent with the idea that the Fur monomer is an apo-form Fur (10, 17, 19). The Zn(II)-bound Fur homodimer contained about 1.02 ± 0.10 Zn and less than 0.1 Fe or sulfide atoms per Fur monomer, suggesting that the Zn(II)-bound Fur homodimer binds one Zn(II) only. The results for metal contents are summarized in Table 1.

**Table 1:** Iron, sulfide, and zinc contents of purified Fur proteins

*E. coli* coli Fur protein	Fe/Fur monomer^a^	S/Fur monomer^b^	Zn/Fur monomer^a^
Apo-Fur (purified from M9)	0.04 ± 0.03	0.05 ± 0.04	0.07 ± 0.03
[2Fe–2S]-bound Fur (fraction 21)	1.86 ± 0.30	1.60 ± 0.40	0.08 ± 0.03
Apo-Fur (fraction 24)	0.05 ± 0.02	0.07 ± 0.05	0.06 ± 0.02
Zn-bound Fur (fraction 21)	0.08 ± 0.05	0.09 ± 0.05	1.02 ± 0.10

Note that the ratios were calculated based on iron, sulfide, and zinc atoms per Fur monomer.

a Fe and Zn contents were obtained from the ICP–MS measurements.

b Sulfur contents were determined using the Siegel’s method (37). The data are averages ± standard deviations from three independent experiments (n = 3).

### The [2Fe–2S] cluster–bound Fur homodimer and the Zn(II)-bound Fur homodimer have similar binding activity for the Fur-box *in vitro*

To compare the DNA-binding activity of purified Fur homodimers and monomers, we used the restriction site protection assay as described previously (26). Briefly, the promoter region of the operon *iucABCD*, which encodes the enzymes for biosynthesis of siderophore aerobactin, has a consensus Fur-box sequence (5′-GAGAATCATTAGCATTCGC-3′), which also contains the restriction HinfI site (5′-GANTC-3′) (4). The promoter region of the operon *iucABCD* was cloned into plasmid pUC19 to create pUC19-*iuc* (26). Binding of an active Fur to the Fur-box protects the HinfI site from being cleaved by HinfI (4) and forms a large DNA fragment of 787 bp (26). This approach was used to determine the Fur-box binding of *E. coli* Fur after NO exposure (21), of the Co(II)-bound *H. pylori* Fur (19), and of the Mn(II)-bound *Aliivibrio salmonicida* Fur (38). In the experiments, pUC19-*iuc* was preincubated with increasing concentrations of the Fur homodimers or monomers, followed by the Hinf1 digestion.

**Figure 2:**
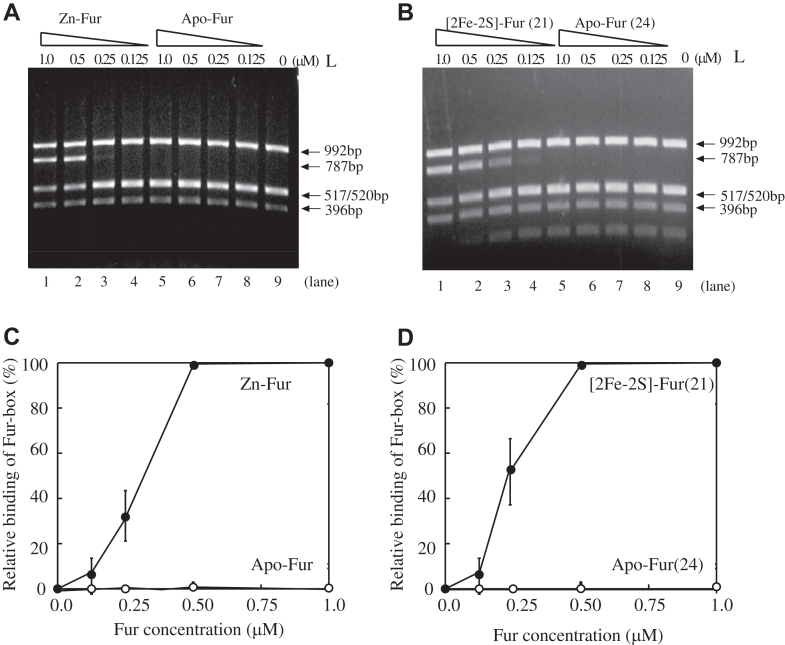
**The Fur-box binding activity of the zinc (Zn)-bound Fur homodimer and the [2Fe–2S] cluster–bound Fur dimer.** pUC19-*iuc* (3.2 nM) was preincubated with increasing concentrations of Fur homodimer or monomers, followed by digestion with HinfI (1.0 unit) at 37 °C for 10 min. The digested DNA products were separated by 1.5% agarose gel electrophoresis. (*A*), the Fur-box binding activity of the Zn-bound Fur homodimer. Lanes 1 to 4, plasmid pUC19-*iuc* was preincubated with 1.0 μM, 0.5 μM, 0.25 μM, and 0.12 μM Zn-bound Fur homodimer (in concentrations of Fur monomer). Lanes 5 to 8, plasmid pUC19-*inc* was preincubated with 1.0 μM, 0.5 μM, 0.25 μM, and 0.12 μM apo-form Fur monomer purified from *Escherichia coli* cells grown in M9 medium only. Lane 9, no Fur was added before the HinfI digestion. Lane L, molecular markers of DNA. *B,* the Fur-box binding activity of the [2Fe–2S]–bound Fur homodimer. Lanes 1 to 4, plasmid pUC19-*iuc* was preincubated with 1.0 μM, 0.5 μM, 0.25 μM, and 0.12 μM [2Fe–2S]-bound Fur homodimer (in concentrations of Fur monomer). Lanes 5 to 8, plasmid pUC19-*inc* was preincubated with 1.0 μM, 0.5 μM, 0.25 μM, and 0.12 μM apo-form Fur monomer (fraction 24). Lane 9, no Fur was added before the HinfI digestion. Lane L, molecular markers of DNA. *C,* Fur-box binding activity of the Zn-bound Fur homodimer. The intensities of the DNA band at 787 bp shown in (*A*) were quantified using ImageJ and plotted as a function of the Fur concentrations. Data represent the averages ± standard deviations from three independent experiments. *D,* Fur-box binding activity of the [2Fe–2S]-bound Fur homodimer. The intensities of the DNA band at 787 bp shown in (*B*) were quantified using ImageJ and plotted as a function of the Fur concentrations. Data represent the averages ± standard deviations from three independent experiments. Fe–S, iron–sulfur; Fur, ferric uptake regulator.

Figure 2*A* shows that the Zn(II)-bound Fur homodimer was active to bind the Fur-box DNA. About 0.5 μM Zn(II)-bound Fur homodimer was sufficient to protect the Fur-box from being cleaved by Hinf1, which was comparable to that previously reported by others (19, 21, 38). In contrast, apo-form Fur monomer purified from the *E. coli* cells grown in M9 medium had no Fur-box activity (26). Under the same experimental conditions, the [2Fe–2S] cluster–bound Fur homodimer (fraction 21) was also active to bind the Fur-box as described (26), whereas the apo-form Fur (fraction 24) was not active (Fig. 2*B*). The intensity of the protected DNA band was quantified using ImageJ (National Institutes of Health) and plotted in Figure 2, *C* and *D* for the Zn(II)-bound Fur homodimer and the [2Fe–2S] cluster–bound Fur homodimer, respectively. Evidently, the Zn(II)-bound Fur homodimer and the [2Fe–2S] cluster–bound Fur homodimer have a similar binding activity for the Fur-box DNA *in vitro*.

### Fur binds Zn(II) and the [2Fe–2S] cluster at the same binding site (site 3)

**Figure 3:**
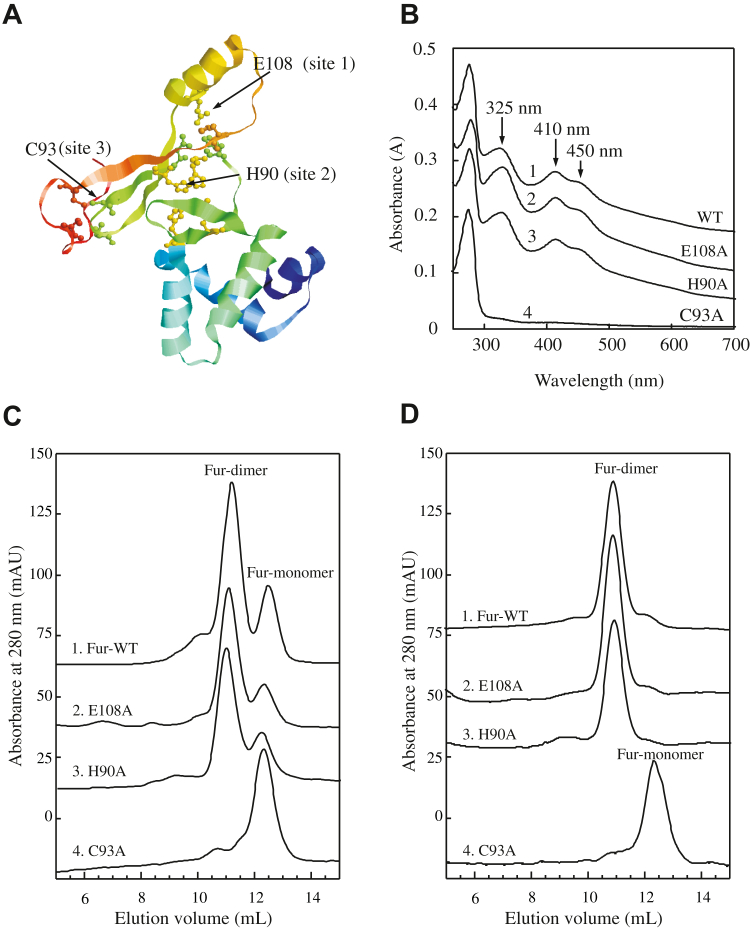
**Fur binds a [2Fe–2S] cluster or zinc (Zn(II)) at the same binding site (site 3).***A,* AlphaFold (AF) model of a full-length *Escherichia coli* Fur with three metal-binding sites. *B,* UV–visible absorption spectra of wildtype Fur (spectrum 1), Fur mutant E108A (site 1) (spectrum 2), H90A (site 2) (spectrum 3), and C93A (site 3) (spectrum 4) purified from the *E. coli* Fur–deleted *E. coli* mutant cells grown in M9 medium supplemented with Fe(NH_4_)_2_(SO_4_)_2_ (4 μM). The purified Fur protein concentration was about 35 μM. Each spectrum was shifted by an absorbance of 0.05 for clarity. *C,* gel filtration profiles of wildtype *E. coli* Fur (trace 1), Fur-E108A (trace 2), Fur-H90A (trace 3), and Fur-C93A (trace 4) purified from *E. coli* Fur–deleted mutant *E. coli* cells grown in M9 medium supplemented with Fe(NH_4_)_2_(SO_4_)_2_ (4 μM). *D,* gel filtration profiles of wildtype *E. coli* Fur (trace 1), Fur-E108A (trace 2), Fur-H90A (trace 3), and Fur-C93A (trace 4) purified from *E. coli* Fur–deleted mutant *E. coli* cells grown in M9 medium supplemented with ZnSO_4_ (4 μM). Fe–S, iron–sulfur; Fur, ferric uptake regulator.

The available crystal structure of *E. coli* Fur only contains the N-terminal region of the amino acid residues 1 to 82 (10). The full-length *E. coli* Fur structure model was obtained using the AlphaFold2 (39). In this model, three metal-binding sites are shown: site 1 (coordinated by His-87, Asp-89, Glu-108, and His-125), site 2 (coordinated by His-33, Glu-81, His-88, and His-90), and site 3 (coordinated by Cys-93 and Cys-96) (10, 12, 17, 19) (Fig. 3*A*). Site 1 was initially described as a regulatory site, whereas site 2 was a structural one (40). However, this proposal was challenged by the mutagenesis study of several Fur proteins (3, 12, 13) and by an *in silico* modeling of *E. coli* Fur (41). Thus, the specific functions of the metal-binding sites in Fur have not been fully defined.

To explore the role of each binding site in the Fur dimerization, we constructed three *E. coli* Fur mutants, in which Glu-108 (site 1), His-90 (site 2), and Cys-93 (site 3) were mutated to Ala, respectively (Fig. 3*A*). Each Fur mutant protein was expressed in the Fur-deleted *E. coli* cells grown in M9 medium supplemented with Fe. Purified Fur mutants and wildtype Fur were then subjected to the UV–visible absorption measurements. Figure 3*B* shows that while mutations of site 1 (E108A) and site 2 (H90A) had no effects on the [2Fe–2S] cluster binding in Fur, mutation of site 3 (C93A) abolished the [2Fe–2S] cluster binding in Fur as reported previously (25, 27). Three Fur mutants and wildtype Fur were then subjected to gel filtration analyses (Fig. 3*C*). Similar to wildtype Fur (trace 1), Fur mutants E108A (site 1) (trace 2) and H90A (site 2) (trace 3) had the Fur homodimer peak and the Fur monomer peak. However, the Fur mutant C93A (site 3) (trace 4) had only the Fur monomer peak, suggesting that the Fur mutant C93A that fails to bind a [2Fe–2S] cluster at site 3 does not dimerize in *E. coli* cells.

Three Fur mutants were also expressed in the Fur-deleted *E. coli* cells grown in M9 medium supplemented with Zn(II), purified, and followed by gel filtration analyses. Figure 3*D* shows that while wildtype Fur (trace 1), Fur mutants E108A (site 1) (trace 2), and H90A (site 2) (trace 3) had a single Zn(II)-bound Fur homodimer peak, the Fur mutant C93A (site 3) (trace 4) had the single apo-form Fur monomer peak. The metal analysis showed that the mutations of E108A (site 1) and H90A (site 2) did not change the Zn(II) binding or the [2Fe–2S] cluster binding in *E. coli* Fur, whereas the mutation of C93A (site 3) completely eliminated the Zn(II) binding or the [2Fe–2S] cluster binding in Fur (data not shown). The results agree with the previous reports showing that *E. coli* Fur binds the tight Zn(II) at site 3 *via* the cysteine residues (10, 16, 17, 18, 19). Taken together, *E. coli* Fur binds the [2Fe–2S] cluster or Zn(II) at the same binding site (site 3) and forms a homodimer.

### The [2Fe–2S] cluster–bound Fur homodimer dissociates into Fur monomers when the [2Fe–2S] cluster disassembles upon reduction

**Figure 4:**
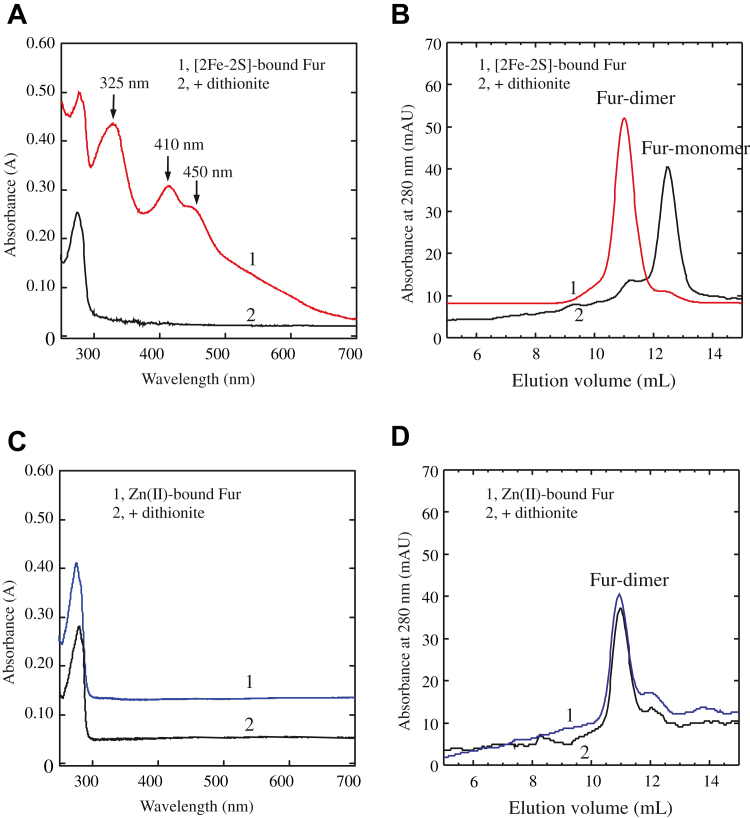
**The [2Fe-2S] cluster–bound Fur homodimer dissociates into monomers when the [2F–2S] cluster is disrupted.***A,* UV–visible absorption spectra of the [2Fe–2S] cluster–bound Fur homodimer before and after removal of the cluster. Spectrum 1, the [2Fe–2S] cluster–bound Fur homodimer (purified from the gel filtration). Spectrum 2, the Fur after being reduced with sodium dithionite (4 mM). The concentrations of the Fur proteins were 50 μM. *B,* gel filtration profiles of the [2Fe–2S]-bound Fur homodimer before and after reduction with sodium dithionite. Trace 1, before reduction with sodium dithionite. Trace 2, after reduction with sodium dithionite. *C,* UV–visible absorption spectra of the Zn(II)-bound Fur homodimer before and after reduction with sodium dithionite. Spectrum 1, the Zn(II)-bound Fur homodimer purified from the gel filtration. Spectrum 2, the Zn(II)-bound Fur after being reduced with sodium dithionite (4 mM). The concentrations of the Zn(II)-Fur proteins were 50 μM. Spectrum 2 was shifted by an absorbance of 0.1. *D,* gel filtration profiles of the Zn(II)-Fur homodimer before and after reduction with sodium dithionite. Trace 1, before reduction with sodium dithionite. Trace 2, after reduction with sodium dithionite. The data are representative of three independent experiments. Fe–S, iron–sulfur; Fur, ferric uptake regulator.

When the [2Fe–2S] cluster–bound Fur homodimer (fraction 21) was reduced with sodium dithionite, Fur quickly lost the [2Fe–2S] cluster to form an apo-form Fur (Fig. 4*A*), as described previously (27, 28). The reduced Fur proteins were then subjected to gel filtration analyses. Figure 4*B* shows that when the [2Fe–2S] cluster was disassembled upon reduction, the Fur homodimer dissociated into Fur monomers. Disruption of the [2Fe–2S] cluster in Fur was reversible, as reconstitution of the [2Fe–2S] cluster in apo-form Fur (Fig. S2*A*) largely restored the dimerization of Fur (Fig. S2*B*) and its Fur-box binding activity (Fig. S2*C*).

In the parallel experiments, when the Zn(II)-bound Fur homodimer was reduced with sodium dithionite, no change of the absorption spectrum (Fig. 4*C*) or the dimeric state (Fig. 4*D*) of the Zn(II)-bound Fur homodimer was observed. The Fur-box binding activity of the Zn(II)-bound Fur homodimer also remained the same after reduction with sodium dithionite. Thus, unlike the [2Fe–2S] cluster–bound Fur homodimer, which becomes an apo-form monomer upon reduction of the cluster, the Zn(II)-bound Fur homodimer is redox inactive.

### The Fe–S cluster scaffold protein IscU is required for the [2Fe–2S] cluster–mediated dimerization of Fur and dispensable for the Zn(II)-mediated dimerization of Fur *in vivo*

**Figure 5:**
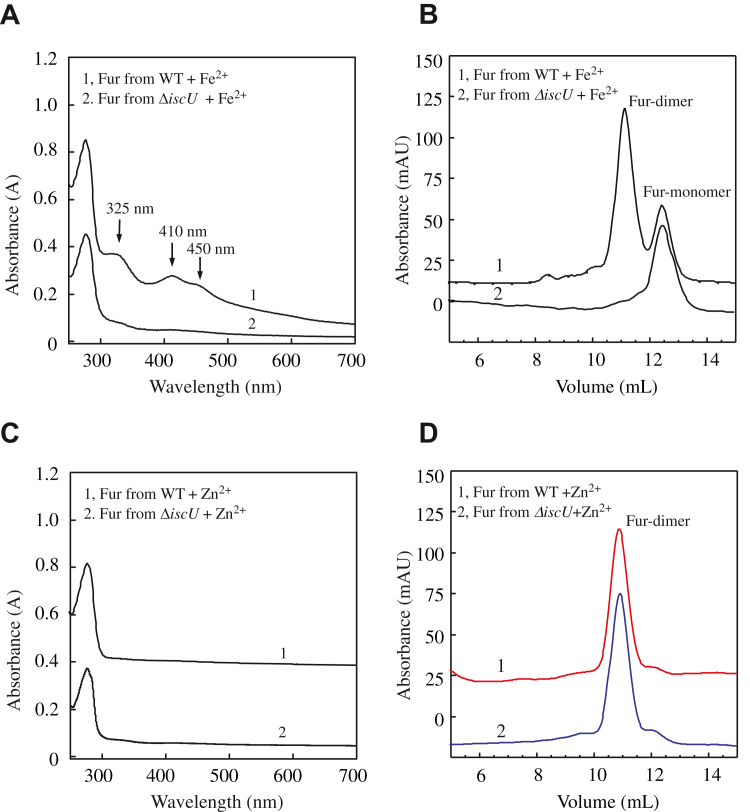
**IscU is essential for the [2Fe–2S] cluster–mediated dimerization of Fur and dispensable for the Zn(II)-mediated dimerization of Fur in *Escherichia coli* cells.***A,* Fur was expressed in wildtype *E. coli* or the IscU-deleted mutant cells grown in M9 medium supplemented with Fe(NH_4_)_2_(SO_4_)_2_ (4 μM). Spectrum 1, UV–visible absorption spectrum of Fur (80 μM) purified from wildtype *E. coli* cells. Spectrum 2, UV–visible absorption spectrum of Fur (80 μM) purified from the IscU-deleted *E. coli* mutant cells. *B,* gel filtration profiles of purified Fur proteins. Trace 1, Fur purified from wildtype *E. coli* cells grown in M9 medium supplemented with Fe(NH_4_)_2_(SO_4_)_2_ (4 μM). Trace 2, Fur purified from the IscU-deleted *E. coli* mutant cells grown in M9 medium supplemented with Fe(NH_4_)_2_(SO_4_)_2_ (4 μM). The results are representative of three independent experiments. *C,* Fur was expressed in *E. coli* wildtype or the IscU-deleted mutant cells grown in M9 medium supplemented with ZnSO_4_ (4 μM). Spectrum 1, UV–visible absorption spectrum of the Fur (80 μM) purified from wildtype *E. coli* cells. Spectrum 2, UV–visible absorption spectrum of Fur (80 μM) purified from the IscU-deleted *E. coli* mutant cells. *D,* gel filtration profiles of purified Zn(II)-bound Fur proteins. Trace 1, Fur purified from wildtype *E. coli* cells grown in M9 medium supplemented with ZnSO_4_ (4 μM). Trace 2, Fur purified from the IscU-deleted *E. coli* mutant cells grown in M9 medium supplemented with ZnSO_4_ (4 μM). The results are representative of three independent experiments. Fe–S, iron–sulfur; Fur, ferric uptake regulator; Zn, zinc.

The [2Fe–2S] cluster assembly in Fur requires the Fe–S cluster assembly machinery, as deletion of the Fe–S cluster assembly scaffold protein IscU prevents the [2Fe–2S] cluster formation in Fur in *E. coli* cells grown in M9 medium supplemented with Fe (29) (Fig. 5*A*). When the Fur purified from the IscU-deleted *E. coli* mutant cells was subjected to gel filtration analysis, it had only the Fur monomer peak (Fig. 5*B*), suggesting that IscU is required for the [2Fe–2S] cluster assembly in Fur and dimerization of Fur in *E. coli* cells.

Fur was also expressed in the IscU-deleted *E. coli* mutant cells grown in M9 medium supplemented with Zn (Fig. 5*C*). The gel filtration analysis showed that purified Fur was the Zn(II)-bound homodimer (Fig. 5*D*), suggesting that deletion of IscU had no effects on the Zn(II) binding in Fur and dimerization of Fur in *E. coli* cells. Thus, Fur binds the [2Fe–2S] cluster and Zn(II) in distinct mechanisms in *E. coli* cells.

### Zn(II) competes for the [2Fe–2S] cluster binding in Fur *in vivo*

**Figure 6:**
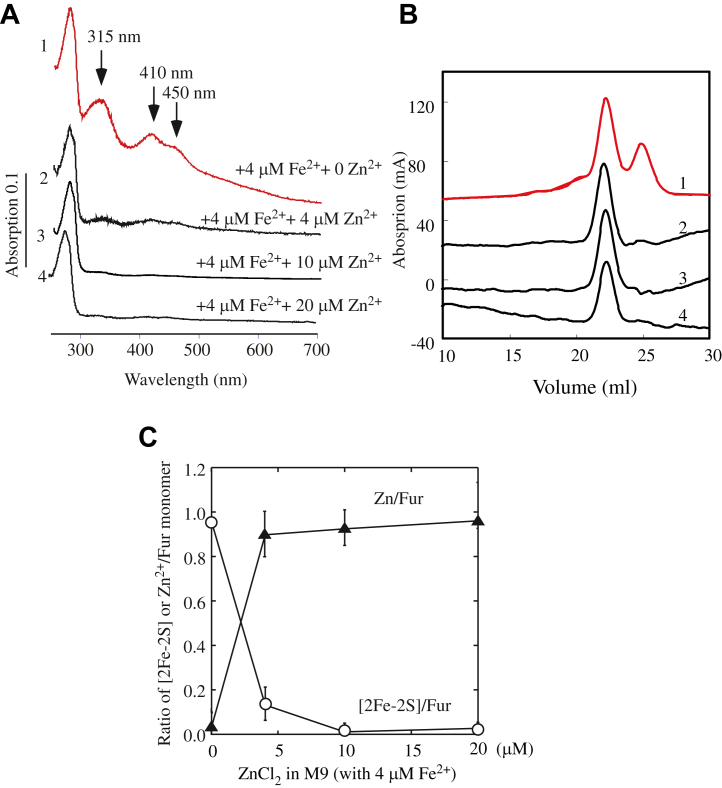
**Zinc (Zn(II)) competes for the [2Fe–2S] cluster binding site in Fur *in vivo*.***A,* UV–visible absorption spectra of purified Fur proteins. Fur proteins were purified from *Escherichia coli* cells grown in M9 medium supplemented with 4 μM Fe(NH_4_)_2_(SO_4_)_2_ and 0 μM (spectrum 1), 4 μM (spectrum 2), 10 μM (spectrum 3), and 20 μM (spectrum 4) ZnSO_4_, respectively. The absorption peaks at 315 nm, 410 nm, and 450 nm represent the [2Fe–2S] cluster in Fur. *B,* gel filtration profiles of purified Fur proteins. Purified Fur proteins (from *A*) were subjected to the gel filtration column. The Fur proteins were purified from *E. coli* cells grown in M9 medium supplemented with Fe(NH_4_)_2_(SO_4_)_2_ (4 μM) and 0 μM (trace 1), 4 μM (trace 2), 10 μM (trace 3), or 20 μM (trace 4) ZnSO_4_. *C,* the [2Fe–2S] cluster and Zn(II) contents in Fur proteins. The ratios of the [2Fe–S] cluster and Zn(II) per Fur monomer in the purified Fur homodimer were analyzed and plotted as a function of the ZnSO_4_ concentrations in M9 medium supplemented with 4 μM Fe(NH_4_)_2_(SO_4_)_2_. The results are averages ± standard deviations from three independent experiments. Fe–S, iron–sulfur; Fur, ferric uptake regulator.

The shared binding (site 3) of the Zn(II) and the [2Fe–2S] cluster in *E. coli* Fur (Fig. 3) suggests that Zn(II) may compete with the [2Fe–2S] cluster binding in Fur. To test this idea, Fur was expressed in *E. coli* cells grown in M9 medium supplemented with Fe(II) (4 μM) and increasing concentrations of Zn(II) (0, 4, 10, and 20 μM). Purified Fur proteins were then subjected to the UV–visible absorption measurements, followed by gel filtration analysis. When *E. coli* cells were grown in M9 medium supplemented with Fe(II) (4 μM) only, purified Fur had both the [2Fe–2S] cluster–bound homodimer and apo-form monomer, as described in Figure 1. The addition of Zn(II) (4 μM) to M9 medium largely eliminated the [2Fe–2S] cluster binding in Fur (Fig. 6*A*) and shifted the Fur monomer to the Fur homodimer (Fig. 6*B*). The Fe and Zn content analysis of the Fur homodimer showed that about 85% of the Fur was the Zn(II)-bound homodimer. Increase of the Zn(II) concentrations in M9 medium further increased the Zn(II)-bound Fur homodimer with concomitant decrease of the [2Fe–2S] cluster–bound Fur homodimer (Fig. 6*C*), showing that Zn(II) can effectively compete for the [2Fe–2S] cluster binding in Fur in *E. coli* cells. It should be pointed out that the competition of Zn(II) and the [2Fe–2S] cluster for the same binding site in proteins is not unprecedented, as Zn(II) and the [2Fe–2S] cluster have the similar ligand coordination (42).

## Discussion

Here, we report that the global Fe regulator Fur binds a [2Fe–2S] cluster or Zn(II) per monomer when Fur is expressed in *E. coli* cells grown in M9 medium supplemented with Fe or Zn, respectively. The Zn(II)-bound Fur (10, 16, 17) and the [2Fe–2S] cluster–bound Fur are homodimers (Fig. 1) and equally active in binding the Fur-box *in vitro* (Fig. 2). The ICP–MS measurements show that the [2Fe–2S] cluster–bound Fur homodimer binds only one [2Fe–2S] cluster per monomer and no other transition cations, and that the Zn(II)-bound Fur homodimer binds only one Zn(II) per monomer (Table 1). The site-directed mutagenesis studies further reveal that Fur binds the [2Fe–2S] cluster or Zn(II) at the same binding site (site 3) *via* the cysteine residues (Fig. 3). Nevertheless, unlike the [2Fe–2S] cluster–bound Fur homodimer, which releases the [2Fe–2S] cluster upon reduction (27) and dissociates to monomers, the Zn(II)-bound Fur homodimer is stable upon reduction (Fig. 4). While the [2Fe–2S] cluster binding in Fur requires the Fe–S cluster assembly scaffold protein IscU (29), deletion of IscU has no effects on the Zn(II) binding in Fur and dimerization of Fur in *E. coli* cells (Fig. 5), indicating that Fur binds the [2Fe–2S] cluster and Zn(II) in distinct mechanisms. Furthermore, Zn(II) can effectively compete for the [2Fe–2S] cluster binding in Fur in *E. coli* cells (Fig. 6). The results led us to propose that the binding of a [2Fe–2S] cluster, like the binding of Zn(II), at site 3 drives dimerization of Fur and turns on the Fur-box binding activity of Fur in *E. coli* cells.

Oligomerization of transcription factors is an important regulatory mechanism for modulating their DNA-binding specificity and affinity. For example, the *E. coli* anaerobic transcriptional regulator FNR controls gene expression *via* the transition from FNR monomer to FNR homodimer (43, 44, 45). Under anaerobic conditions, FNR assembles a [4Fe–4S] cluster and forms a homodimer to bind its target DNA. Under aerobic conditions, the [4Fe–4S] cluster is degraded by oxygen, resulting in FNR monomer, which has no DNA-binding activity (45). Degradation of the [4Fe–4S] cluster by oxygen propagates a conformational signal that causes monomerization by disrupting the specific interactions between two FNR monomers (43). While the crystal structure of the [2Fe–2S] cluster–bound Fur is currently not available, it may be envisioned that dimerization of Fur monomers is similarly driven by binding of the [2Fe–2S] cluster through specific protein–protein interactions between two monomers.

The previous hypothesis that Fur binds Fe(II) to become an active repressor (5, 6, 7) was primarily based on the observations that apo-form Fur binds various transition cations, including Fe(II), Co(II), Mn(II), and Zn(II) *in vitro* (4, 8, 9). While no Fe(II)-bound Fur had been identified *in vivo*, the Fur protein purified from *E. coli* cells grown in LB medium contained two Zn(II) atoms, a tightly bound Zn(II), and a loosely bound Zn(II) (10, 16, 17). Chemical modification and MS analysis revealed that *E. coli* Fur binds a tight Zn(II) at site 3 *via* the cysteine residues and forms a homodimer (17, 18, 19). When an excess amount of metal chelator EDTA (100 mM) was included in the protein purification solution, isolated *E. coli* Fur was a metal-free apo-form monomer (10, 17). However, adding Zn(II) to the purified Fur monomers did not lead to dimerization of Fur. Only when the Fur monomer was first reduced with dithiothreitol, the addition of a stoichiometric amount of Zn(II) resulted in dimerization of Fur (10, 17). These results suggested that *E. coli* Fur binds a tight Zn(II) at site 3 *via* the cysteine residues and a loose Zn(II) at site 2 *via* histidine and glutamate acid residues (10, 16, 17).

**Figure 7:**
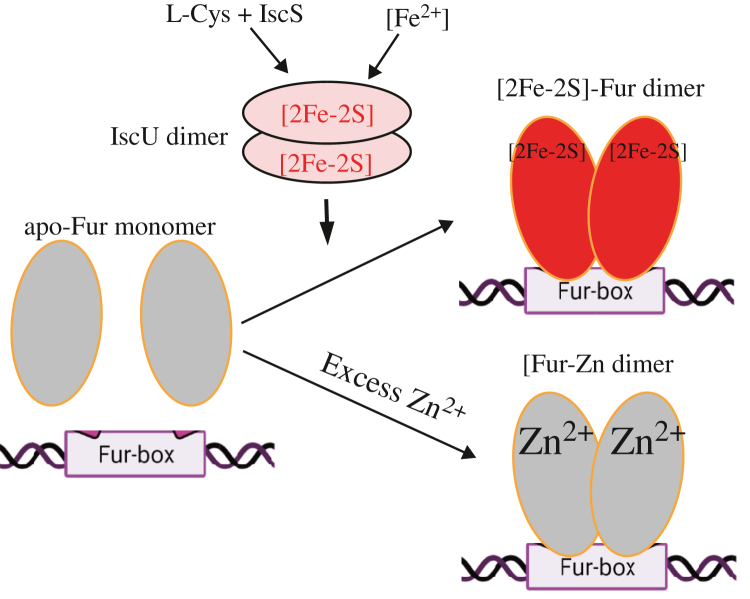
**A proposed model for regulation of *Escherichia coli* Fur in response to elevation of intracellular-free iron and zinc (Zn) contents.** When intracellular-free iron content is scarce, apo-form Fur is a monomer and does not bind the Fur-box. When intracellular-free iron content is elevated, Fur assembles a [2Fe–2S] cluster, forms a homodimer, and binds the Fur-box. When intracellular Zn(II) concentration is elevated, Zn(II) competes for the [2Fe–2S] cluster binding site in Fur, prevents Fur to sense intracellular-free iron content, and disrupts the regulation of intracellular iron homeostasis. Fe–S, iron–sulfur; Fur, ferric uptake regulator.

Unlike LB medium, which contains significant amounts of Zn(II) and other transition cations, M9 medium has the defined ingredients and limited amounts of transition metal cations (46). When *E. coli* Fur is expressed in *E. coli* cells grown in M9 medium, purified Fur is an apo-form protein (26), which is a monomer (Fig. 1), as described previously (10, 17). When *E. coli* Fur is expressed in the *E. coli* cells grown in M9 medium supplemented with Zn(II) (4 μM), Fur binds one Zn(II) atom per monomer and forms a homodimer (Fig. 1*B*), similar to that purified from the *E. coli* cells grown in LB medium (10, 16, 17). When *E. coli* Fur is expressed in *E. coli* cells grown in M9 medium supplemented with Fe(II) (4 μM), about 38% of Fur is a [2Fe–2S] cluster–bound Fur homodimer (26) (Fig. 1, *C* and *D*). Further increase of Fe in M9 medium does not increase the amount of the [2Fe–2S] cluster–bound Fur homodimer (26). Importantly, the ICP–MS measurements show that the purified [2Fe–2S] cluster–bound *E. coli* Fur homodimer binds only one [2Fe–2S] cluster and no Zn(II) per monomer and that the Zn(II)-bound Fur homodimer binds only one Zn(II) per monomer. The site-directed mutagenesis studies further indicate that the mutation of site 3 (Cys-93 to Ala) results in a Fur mutant that fails to bind the [2Fe–2S] cluster or Zn(II) (Fig. 5). Thus, the binding of a [2Fe–2S] cluster or Zn(II) at site 3 (*via* the cysteine residues) is necessary and sufficient for dimerization of *E. coli* Fur (Fig. 7). This view is consistent with the previous mutagenesis studies (19, 47, 48). Nevertheless, the intactness of the metal-binding sites 1 and 2 may also be crucial for the structure and function of Fur (10, 12, 13, 14). Although we find no Fe or Zn(II) binding in site 1 and site 2 in *E. coli* Fur purified from the *E. coli* cells grown in M9 medium supplemented with 4 μM Fe or Zn (Table 1), that does not exclude the possibility that Fur may bind Fe(II) or Zn(II) at site 2 in cells. Interestingly, some Fur homologs, such as *Pseudomonas aeruginosa* Fur, do not have the corresponding cysteine residues at site 3 (49). How these Fur proteins respond to the elevation of intracellular-free Fe content in these bacteria remains to be investigated.

Although both the [2Fe–2S] cluster–bound Fur and the Zn(II)-bound Fur are homodimers and have similar Fur-box binding activity *in vitro* (Fig. 2), it is likely that they have distinct binding activities for Fur-boxes of specific Fur-regulated genes in cells. Indeed, the Zn(II)-bound Fur appears to be inactive to repress the Fur-regulated genes in *E. coli* cells (4, 16). Recent studies also showed that excess Zn(II) transiently upregulates Fe-uptake genes and downregulates Fe-storage genes and thereby increases the cellular Fe quota in *E. coli* cells (50). Perhaps, the Zn(II)-bound Fur binds the Fur-box of the gene *fur* promoter and represses the expression of Fur itself (5). Regardless, the binding of Zn(II) at site 3 in Fur effectively prevents the [2Fe–2S] cluster binding in Fur (Fig. 6) and disrupts signaling of intracellular Fe homeostasis in *E. coli* cells.

In summary, we propose that when the amounts of other transition cations (such as Zn(II)) are limited (*e.g.*, in M9 medium), *E. coli* Fur assembles a [2Fe–2S] cluster at site 3 *via* the Fe–S assembly machinery in response to elevation of intracellular free Fe content and forms a homodimer to regulate intracellular Fe homeostasis. When the amounts of other transition cations are high (*e.g.*, in LB medium), they compete for the [2Fe–2S] cluster binding in Fur and disregulate the intracellular Fe homeostasis in cells (Fig. 7).

## Experimental procedures

### Protein purification

Plasmid pBAD expressing wildtype *E. coli* Fur or the Fur mutants (Fur-C93A, Fur-H90A, or Fur-E108A) was introduced into the *E. coli* Fur–deleted mutant cells as described (26) or the *E. coli* IscU–deleted mutant cells as described (29). Overnight *E. coli* cultures were inoculated in 1:100 dilution in freshly prepared M9 medium supplemented with 20 amino acids (20 μg/ml each), thiamine (1.0 μg/ml), glycerol (0.4%), Fe(NH_4_)_2_(SO_4_)_2_ (4.0 μM), and ampicillin (100 μg/ml). When the cells were grown to an absorbance of 0.6 at 600 nm at 37 °C under aerobic growth conditions, protein expression was induced by adding l-arabinose (0.04%). The cells were grown for 3 more hours before *E. coli* Fur was purified as described previously (25). The purity of purified Fur proteins was more than 90%, as judged by electrophoresis analysis on a 15% polyacrylamide gel containing SDS followed by staining with Coomassie Blue. The concentration of purified *E. coli* Fur and Fur mutants was measured at 280 nm after the Fe–S cluster in the proteins was removed by adding HCl (20 mM). UV–visible absorption spectra of *E. coli* Fur and Fur mutants were recorded in a Jasco V-750 UV–visible absorption spectrometer at room temperature. The extinction coefficient of 5.6 mM^−1^ cm^−1^ at 280 nm was used for calculating the concentrations of purified Fur and the Fur mutants. The protein concentrations of purified Fur and Fur mutants were expressed as Fur monomers.

### Gel filtration analysis

The oligomeric states of purified *E. coli* Fur and Fur mutants were analyzed by gel filtration using the gel filtration column (Superdex 75 10/300GL [Cytiva Co.]) attached to the FPLC system. The flow rate was 0.8 ml/min using a buffer containing NaCl (500 mM) and Tris (20 mM, pH 8.0). The molecular weights were calibrated using gel filtration standards (Bio-Rad Laboratories, Inc) under the same experimental conditions.

### Fe, Zn, and sulfide content analyses of Fur samples

The amounts of Fe and sulfide in Fur protein samples were analyzed according to the Fisher’s method (36) and the Siegel’s method (37), respectively. The Zn(II) content in Fur protein samples was determined using PAR (4-(2-pyridylazo) resorcinol) under denatured conditions. Briefly, *E. coli* Fur (20 μM) was incubated with PAR (50 μM) and urea (6 M) overnight, followed by centrifugation at 13,000 rpm for 10 min. The extinction coefficient of 66 mM^−1^ cm^−1^ at 500 nm of the Zn(II)–PAR complex (51) was used to calculate the Zn(II) content in Fur after subtracting the absorption amplitude of the Fe(II)–PAR complex as described (52). The total Fe and Zn contents in purified Fur homodimers and monomers were also analyzed using the ICP–MS (University of Georgia, Center for Applied Isotope Studies). The results from the ICP–MS measurements and spectrophotometric analyses were similar to each other.

### Removal of the [2Fe–2S] cluster from *E. coli* Fur homodimer

To remove the [2Fe–2S] cluster from *E. coli* Fur dimer, purified Fur homodimer was incubated with freshly prepared sodium dithionite (4 mM) in 20 mM Tris (pH 8.0) at room temperature for 20 min. The Fur protein was then subjected to gel filtration analysis.

### The HinfI site protection assay

The Fur-box binding activity of *E. coli* Fur was also analyzed using the HinfI site protection assay (4) with the following modifications. The Fur-box in the *E. coli iucABCD* promoter (5′-GAGAATCATTAGCATTCGC-3′), which contains the restriction HinfI site (5′-GAATC-3′), was synthesized (GenScript Co) and inserted into plasmid pUC19 *via* BamHI and HindIII sites to create pUC19-*iuc*. Binding of Fur to the Fur-box protects the HinfI site from being cleaved by HinfI (4). For the HinfI site protection assays, pUC19-*iuc* (3.2 nM) was preincubated with Fur proteins (0–1.0 μM) in 10 μl reaction solutions containing MgCl_2_ (2 mM), NaCl (150 mM), bovine serum albumin (0.1 mg/ml), and Tris (20 mM, pH 8.0) for 10 min at room temperature. Restriction enzyme HinfI (1.0 unit) (New England Biolab Co) was then added to the reaction solutions. After incubation at 37 °C for 10 min, the reaction was stopped by adding 2 μl 6× loading buffer (New England Biolab Co). The digested DNA products were separated by 1.5% agarose electrophoresis gel containing ethidium bromide (0.1 μg/ml) in 0.5X Tris–acetate–EDTA buffer, run at 120 V for 35 min. The gel images were taken using the Kodak Gel Logic 200 Imaging System. The intensities of the DNA bands on the agarose gel images were quantified using ImageJ.

### Statistical analysis

All data are expressed as means ± standard deviations from at least three independent experiments.

## Data availability

All data generated and analyzed in the present study are included in the article. Raw data are available on request.

## Supporting information

This article contains supporting information.

## Conflict of interest

The authors declare that they have no conflicts of interest with the contents of this article.
