# Supplementary material for: Binding of a [2Fe–2S] cluster drives dimerization of ferric uptake regulator (Fur) in Escherichia coli
Source: J Biol Chem. 2025 Sep 11;301(10):110702. doi: 10.1016/j.jbc.2025.110702 (PMC12538065; doi:10.1016/j.jbc.2025.110702)
Supplement: Supporting Figures 1 and 2 [file mmc1.pdf]

Supplemental Figure 1

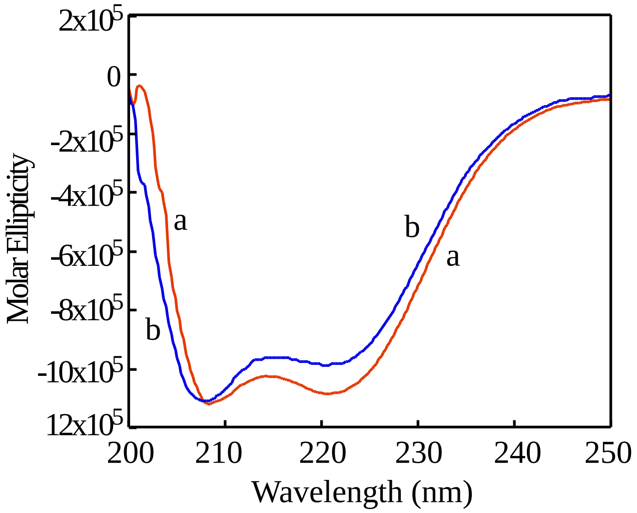

Supplemental Figure 1. Circular Dichroism (CD) spectra of the [2Fe-2S] cluster-bound Fur homodimer and apo-form Fur monomer. The circular dichroism (CD) spectra of *E. coli* Fur were recorded in a Jasco J-815 CD spectrometer at room temperature. The fraction 21 (the [2Fe-2S] cluster-bound Fur homodimer, red trace) and fraction 24 (apo-form Fur monomer, blue trace) (30 μM of Fur monomer each) were dissolved in phosphate buffer (pH 7.4).

Supplemental Figure 2

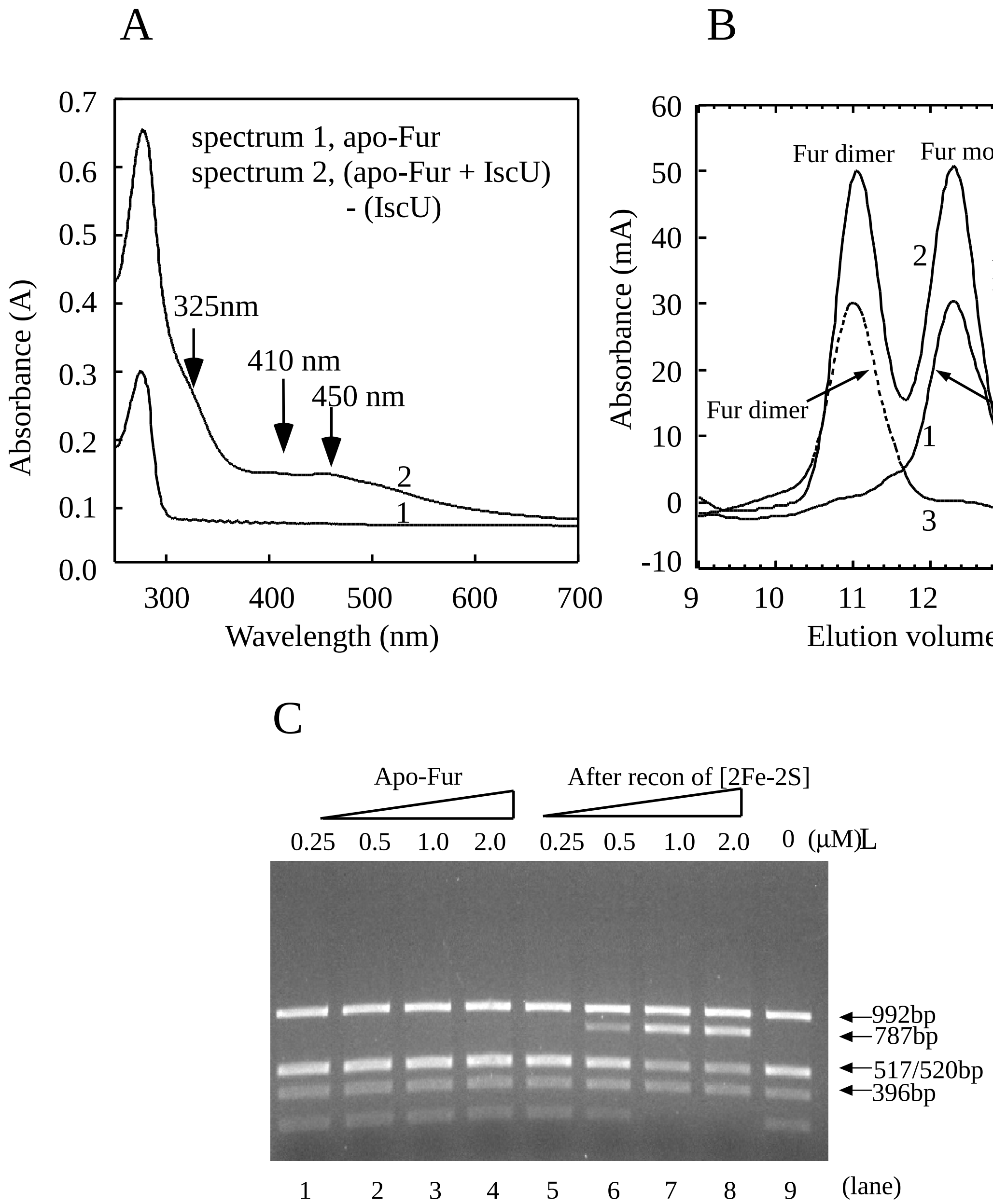

Supplemental Figure 2. Fur forms a homodimer upon reconstitution with a [2Fe-2S] cluster. A), UV-Vis absorption spectra of apo-form Fur before and after reconstitution with the [2Fe-2S] cluster. Spectrum 1, apo-form Fur (50 µM). Spectrum 2, the net spectrum of apo-form Fur after reconstitution with IscU (50 µM), $Fe(NH_4)_2(SO_4)_2$ (1 mM), L-cysteine (1 mM), and cysteine desulfurase (IscS) (1 µM) (at room temperature for 20 min) minus the same reconstitution solution but without apo-form Fur. **B)**, Gel filtration profiles of apo-form Fur (trace 1), apo-form Fur after the reconstitution (trace 2), and the [2Fe-2S] cluster-bound Fur homodimer (trace 3). **C),** the restriction site protection assay. Lanes 1-4, plasmid pUC19-iuc was pre-incubated with 0.25, 0.5, 1.0, and 2.0 µM apo-form Fur. Lanes 5-8, plasmid pUC19-inc was pre-incubated with 0.25, 0.5, 1.0, and 2.0 µM Fur Fur after the reconstitution. Lane 9, no Fur was added before the *Hin*fI digestion. Lane L, molecular marks of DNA. The data are representative of three independent experiments.
